# Neurodevelopmental impairment induced by prenatal valproic acid exposure shown with the human cortical organoid-on-a-chip model

**DOI:** 10.1038/s41378-020-0165-z

**Published:** 2020-07-13

**Authors:** Kangli Cui, Yaqing Wang, Yujuan Zhu, Tingting Tao, Fangchao Yin, Yaqiong Guo, Haitao Liu, Fei Li, Peng Wang, Yuejun Chen, Jianhua Qin

**Affiliations:** 1 Division of Biotechnology, CAS Key Laboratory of SSAC, Dalian Institute of Chemical Physics, Chinese Academy of Sciences, 457 Zhongshan Road, Dalian, 116023 China; 2 University of Chinese Academy of Sciences, Beijing, 100049 China; 3 CAS Center for Excellence in Brain Science and Intelligence Technology, Chinese Academy of Sciences, Shanghai, 200031 China; 4 Institute for Stem Cell and Regeneration, Chinese Academy of Sciences, Beijing, China

**Keywords:** Chemistry, Structural properties, Chemistry, Structural properties

## Abstract

Prenatal exposure to environmental insults can increase the risk of developing neurodevelopmental disorders. Administration of the antiepileptic drug valproic acid (VPA) during pregnancy is tightly associated with a high risk of neurological disorders in offspring. However, the lack of an ideal human model hinders our comprehensive understanding of the impact of VPA exposure on fetal brain development, especially in early gestation. Herein, we present the first report indicating the effects of VPA on brain development at early stages using engineered cortical organoids from human induced pluripotent stem cells (hiPSCs). Cortical organoids were generated on micropillar arrays in a controlled manner, recapitulating the critical features of human brain development during early gestation. With VPA exposure, cortical organoids exhibited neurodevelopmental dysfunction characterized by increased neuron progenitors, inhibited neuronal differentiation and altered forebrain regionalization. Transcriptome analysis showed new markedly altered genes (e.g., KLHL1, LHX9, and MGARP) and a large number of differential expression genes (DEGs), some of which are related to autism. In particular, comparison of transcriptome data via GSEA and correlation analysis revealed the high similarity between VPA-exposed organoids with the postmortem ASD brain and autism patient-derived organoids, implying the high risk of autism with prenatal VPA exposure, even in early gestation. These new findings facilitate a better understanding of the cellular and molecular mechanisms underlying postnatal brain disorders (such as autism) with prenatal VPA exposure. This established cortical organoid-on-a-chip platform is valuable for probing neurodevelopmental disorders under environmental exposure and can be extended to applications in the study of diseases and drug testing.

## Simplified brain models enable effective drug testing

Miniaturized 3D models of the developing human brain provide a unique opportunity to assess the effects of a widely-used drug for neurological disorders on early-stage fetuses. Evidence suggests that valproic acid, a common treatment for epilepsy and bipolar disorder, may undermine healthy brain development in the first trimester of pregnancy. These effects have proven difficult to study, but researchers led by Jianhua Qin of the Dalian Institute of Chemical Physics have demonstrated that human cortical ‘organoids’ model offer valuable insights into this question. These stem cell-derived complex structures mirror key features of the fetal brain, by generating arrays of these organoids, the researchers could measure how valproic acid affects gene expression, cell growth, and maturation. They identified early signatures of abnormal development, supporting further use of this model to study the drug’s impact during pregnancy.

## Introduction

Prenatal exposure to environmental chemicals and pollutants can affect an individual’s lifelong health^1^. During early pregnancy, the developing brain is particularly vulnerable to environmental insults. Valproic acid (VPA) is a typical antiepileptic drug, and pharmacological treatment for a series of brain disorders, including bipolar disorder and epilepsy^2,3^. VPA administration during pregnancy can trigger neurodevelopmental disorders, such as autism and schizophrenia, in offspring^4^. Recent studies on prenatal VPA exposure have mainly been based on animal models and traditional 2D cell culture systems^5–9^. However, these models are limited to their representation of dynamic developmental processes in vivo, complex architecture formation and characteristic of the human brain. In addition, present studies on prenatal VPA exposure are mainly concentrated on the late period of pregnancy. Very few studies have been dedicated to the first trimester of gestation, a stage that is highly vulnerable to external stimuli. As such, obtaining a deep understanding of the effects of prenatal VPA administration on fetal brain development remains challenging.

Cortical organoids represent a new class of 3D models with which to study human brain development and neurological diseases^10,11^. These cortical organoids, which are self-organized 3D multicellular clusters derived from human induced pluripotent stem cells (hiPSCs) or human embryonic stem cells (hESCs)^12–16^, recapitulate key architectural and functional characteristics of the fetal brain at early- or even mid-gestation. Although reductionist in nature, cortical organoids have great potential to bridge the gap between cell monolayers and animal models and emerge as a third approach for developmental studies and disease modeling. At present, cortical organoids have been utilized to develop models of a series of neuropsychiatric diseases, including microcephaly^15^, Zika virus infection^17^, and environmental exposure^16,18–20^ and proven to be powerful as a 3D platform in which to investigate psychiatric disease origin and pathology in vitro.

**Fig. 1:**
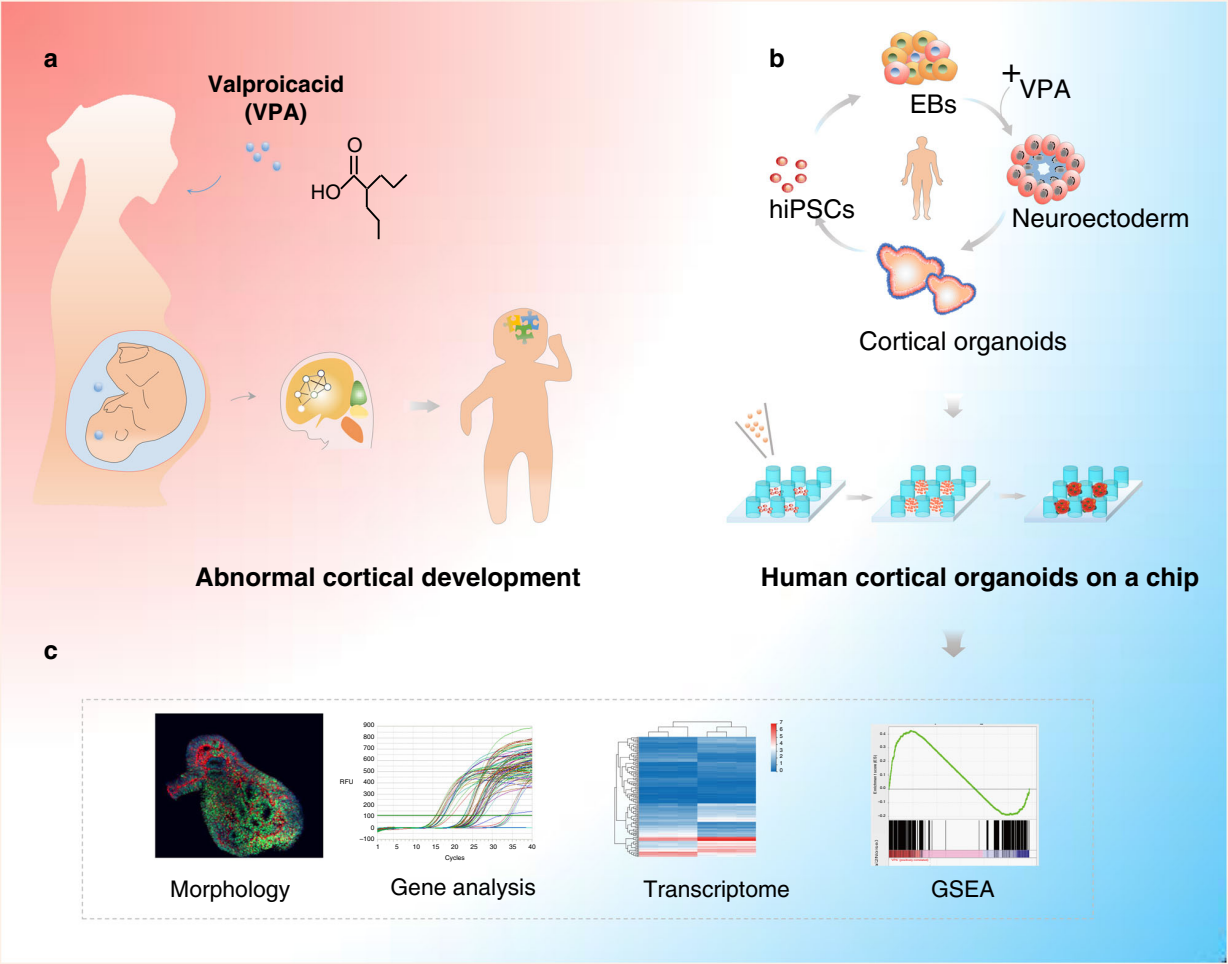
Illustration of the human cortical organoid-on-a-chip model to probe the effects of prenatal VPA exposure on neurodevelopment. **a** Administration of VPA during pregnancy is tightly related to early-stage neurodevelopmental disorders in the fetus, which may give rise to long-term neurobehavioral deficits in offspring. **b** The human cortical organoid-on-a-chip model can recapitulate the developing human brain at early stages during gestation and can be utilized to study abnormal cortical development due to VPA exposure. The process of hiPSC-derived cortical organoid development on a chip and configuration of the cortical organoid-on-a-chip device. **c** The data were analyzed using the different indicated methods.

In this work, we present the first report to probe the effects of VPA exposure on the developing fetal brain at early stages using engineered cortical organoids from hiPSCs. Cortical organoids were simply generated via self-organization on micropillar array devices in a continuous process and a high-throughput manner. Key characteristics of the fetal brain at an early stage, including neurogenesis, forebrain regionalization, and cortical organization, were identified in the resultant cortical organoids. With exposure to VPA, the cortical organoids exhibited significant alterations in neural proliferation, differentiation and maturation, as assessed by immunohistochemical assay and quantitative real-time PCR. Moreover, transcriptome analysis identified a series of newly altered genes and neurodevelopment pathways under VPA exposure. More importantly, by comparison of transcriptomic data via GSEA and correlation analysis, VPA-exposed cortical organoids showed high relevance with the postmortem ASD brain and ASD patient-derived organoids. These results reflected genome-wide changes in cortical organoids exposed to VPA, implying the utility of the established model for exploring neurodevelopmental disorders in offspring exposed to VPA during early gestation (Fig. 1).

## Results

### Characterization of engineered human cortical organoids

**Fig. 2:**
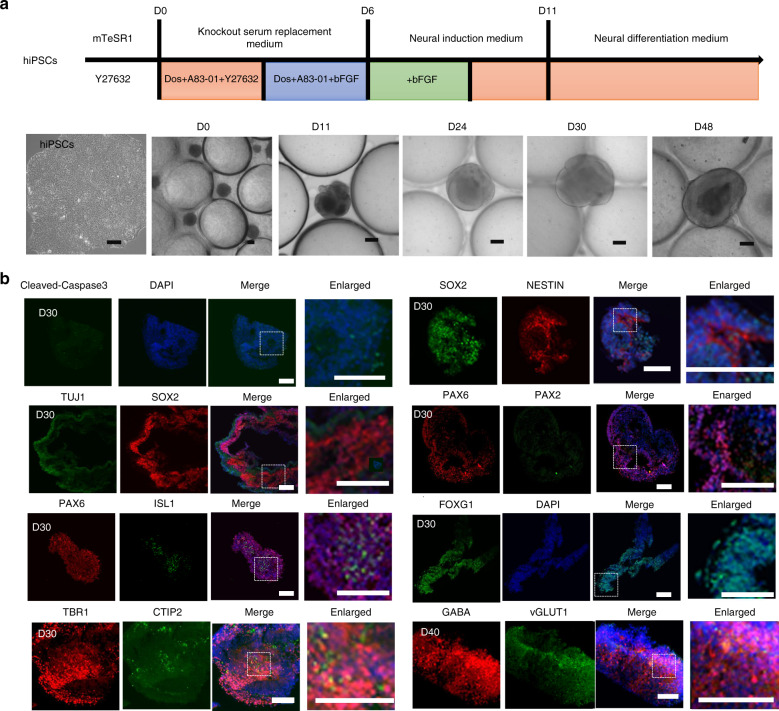
Generation and identification of engineered hiPSC-derived cortical organoids. **a** Schematic diagram and bright-field images of hiPSC-derived cortical organoids developed on a chip. Scale bar = 200 μm. **b** Immunohistochemical image of tissue sections used to detect the apoptosis protein Caspase3, neural progenitor cells (NESTIN and SOX2), differentiated neurons (TUJ1), distinct brain regions (forebrain: FOXG1, PAX6; hindbrain ISL1; developing brain marker PAX2; cortical layer, TBR1 and CTIP2), inhibitory neurons (GABA) and excitatory neurons (vGLUT1) in cortical organoids at 30 and 40 days of differentiation by immunohistochemical analysis. Scale bars = 100 μm.

Here, we generated cortical organoids on micropillar arrays in a simple and controlled manner, which allowed the controlled formation of embryoid bodies (EBs), in situ neural differentiation and the development of cortical organoids from hiPSCs. Specifically, dissociated hiPSCs aggregated into EBs with a consistent size and morphology on the micropillar chips. These EBs were then maintained in neural induction and neural differentiation media in a sequential manner, which created a permissive environment for differentiation and proliferation of the neuroectoderm. Finally, after their culture for further 1–3 weeks, the neuroectodermal spheroids were observed to expand rapidly and differentiate into nearly millimeter-sized cortical organoids (Fig. 2a). To assess cell viability in the cortical organoids, staining for Cleaved-Caspase 3, which is associated with apoptosis, was performed. As shown in Fig. 2b, cortical organoids displayed some cell apoptosis at the tissue level even after their differentiation for 30 days, which validated the availability of our methods.

During brain development, neural stem cells and progenitors from which all neurons and glial cells are generated are made in a process called neurogenesis^21,22^. Therefore, we examined the presence of neocortical neuron populations in cortical organoids. An immunostaining image showed a high proportion of NPCs based on expression of the neural progenitor cell (NPC) markers SOX2 and NESTIN (Fig. 2b). Moreover, the degree of neural differentiation, as characterized by the expression of a neural progenitor marker (SOX2) and a neuron marker (TUJ1), was examined by immunofluorescence assay (Fig. 2b). The results showed neonatal neurons in the periphery of neural progenitor cells, indicating that the cortical organoids had established a well-defined neural identity. As demonstrated in Fig. 2b, the forebrain-specific markers PAX6 and FOXG1 were highly expressed in the cortical organoids. In addition, the hindbrain marker ISL1 was modestly expressed in neighboring regions, mimicking physiological conditions. Furthermore, PAX2, which indicates the developing brain, was also expressed near PAX6 (Fig. 2b). To test the presence of a distinctly layered cortical architecture in the cortical organoids, the pre-plate marker TBR1 and the deep-layer marker CTIP2 were examined (Fig. 2b). Immunofluorescent images showed that early neurons (CTIP2) appeared to be located adjacent to pre-plate TBR1 + neurons, suggesting formation of the cortical plate. In addition, the neuron subtypes inhibitory neurons (GABA) and excitatory neurons (vGLUT1) were detected in cortical organoids at 40 days of differentiation by immunohistochemical analysis.

To further identify gene expression levels at the transcriptome level, we conducted RNA-sequencing analysis of cortical organoids. Differentially expressed genes (DEGs) in cortical organoids compared with hiPSCs were tightly related to neurodevelopment. Gene ontology (GO) analysis showed enrichment of the DEGs in many neuronal function-related pathways (Fig. S1). Therefore, all data revealed the cortical organoids to be a near-physiological cell type with a nearly histological organization, which proved the feasibility of the use of cortical organoids to study human fetal brain development at early stages.

### Impaired neurogenesis in cortical organoids with VPA exposure

**Fig. 3:**
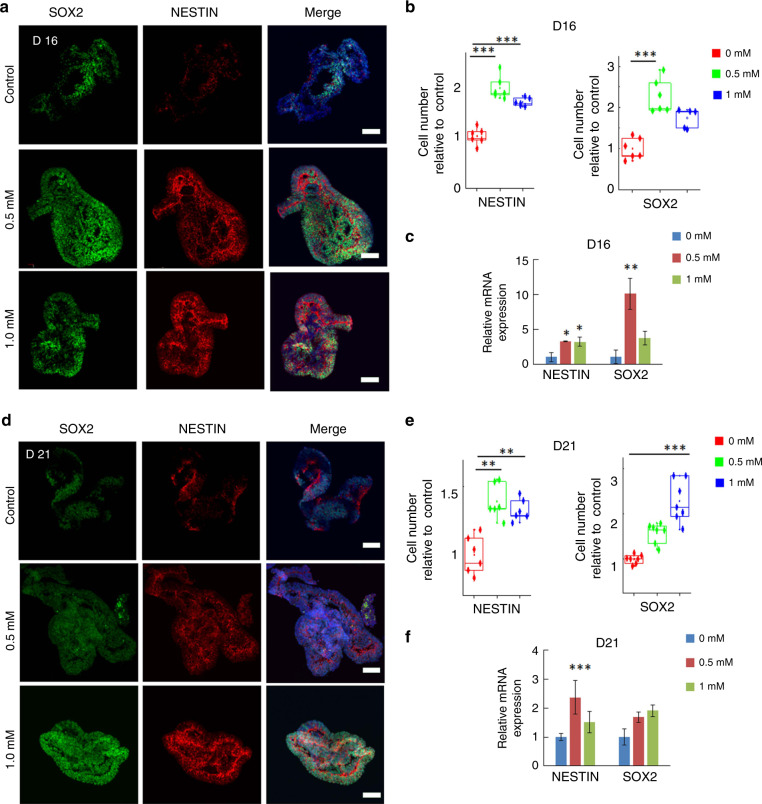
Enhanced neural progenitor cell pool in VPA-exposed cortical organoids. **a**, **d** Immunohistochemistry was performed to identify the percentage of NESTIN + and SOX2 + cells in the cortical organoids exposed to different concentrations of VPA (0.5 mM and 1.0 mM) on day 16 (**a**) and day 21 (**d**). **b**, **e** NESTIN + and SOX2 + cells in cortical organoids exposed to different concentrations of VPA (0.5 mM and 1.0 mM) were quantified on day 16 (**b**) and day 21 (**e**). Five or six images were randomly selected for quantitative analysis on day 16 (**b**) and day 21 (**e**). **c**, **f** Relative mRNA expression of NESTIN and SOX2 in cortical organoids in the absence or presence of VPA (0.5 and 1.0 mM) on day 16 (**c**) and day 21 (**f**) was identified by quantitative real-time PCR. The housekeeping gene β-actin functioned as a normalization control gene. All data are the means of three replicates ± SDs. At least 10 cortical organoids generated on one micropillar chip in each group were randomly examined for quantitative analysis. In addition, three replicate chips for each group were analyzed in each independent experiment. Moreover, more than eight tissue colonies from each sample were randomly analyzed by immunohistochemical staining. The data were analyzed using one-way ANOVA with Bonferroni post-test (*p < 0.05; **p < 0.01; ***p < 0.001; ****p < 0.0001). Scale bar = 100 μm.

To examine the effects of VPA on early fetal brain development, cortical organoids were exposed to VPA. Specifically, the cortical organoids were exposed to VPA at different concentrations (0.5 and 1.0 mM) for 5 and 10 days. Because VPA functions in a clinically relevant range of concentrations <1.0 mM^23^, we selected 0.5 and 1.0 mM for subsequent research. As the cell division of NPCs within the VZ initiates the first step of neuronal development, we first probed the proliferation of neuron progenitor cells (NPCs) with VPA exposure. Immunostaining for the NPC markers SOX2 and NESTIN revealed their higher expression levels in organoids treated with low and high VPA for 5 days compared with control organoids (Fig. 3a), which was confirmed by quantitative analysis of SOX2 + and NESTIN + cells (Fig. 3b). Furthermore, quantitative real-time PCR revealed the induced expression of SOX2 and NESTIN at the mRNA level (Fig. 3c). In agreement with these observations, the cortical organoids revealed a higher proportion of NPCs even after exposure to VPA at different concentrations for 10 days than control organoids did (Fig. 3d–f). These results suggested that VPA could enhance the NPC pool in cortical organoids.

**Fig. 4:**
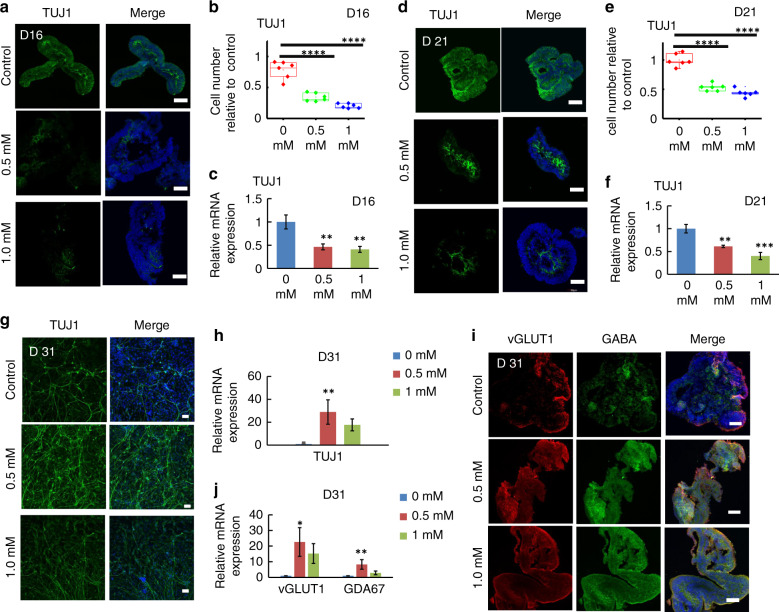
Changes in neuron differentiation and maturation in the presence of VPA. **a**–**f** TUJ1 + cells (**a**, **d**) in cortical organoids treated with different concentrations of VPA (0.5 mM and 1 mM) on day 16 (**a**) and day 21 (**d**) were identified by immunohistochemical analysis. TUJ1 + cells were quantified, and six images were randomly selected for quantitative analysis (**b**, **e**). **c**, **f** The expression of TUJ1 in cortical organoids exposed to VPA at different concentrations on day 16 (**c**) and day 21 (**f**) was identified by real-time PCR. The relative mRNA expression level was calculated by gene expression analysis of the VPA-exposed group relative to the control group. β-Actin acted as a normalization control gene. **g** Immunostaining images of neurons (TUJ1) seeded on Matrigel-coated plates in the absence of VPA on day 21 were shown. **i** Sectioning and immunohistochemical images of excitatory neurons (vGLUT1) and inhibitory neurons (GABA) in the absence of VPA on day 31 were shown. **h**, **j** The expression of TUJ1 (neurons), GAD67 (inhibitory neurons) and vGLUT1 (excitatory neurons) was determined by quantitative real-time PCR. All data are the means of three replicates ± SDs. At least 10 random cortical organoids generated on one micropillar chip in each group were examined for quantitative analysis. In addition, three replicate chips for each group were analyzed in each independent experiment. Moreover, more than eight tissue colonies for each sample were randomly analyzed by immunohistochemical staining. The data were analyzed using a one-way ANOVA with Bonferroni post-test (*p < 0.05; **p < 0.01; ***p < 0.001; ****p < 0.0001). Scale bar = 100 μm.

During development, NPCs give rise to various neuronal lineages; therefore, we explored neuronal differentiation in organoids exposed to VPA. In contrast to NPCs, immunofluorescence staining demonstrated fewer differentiated neurons in organoids exposed to VPA for 5 and 10 days (Fig. 4a–f). Moreover, relative mRNA expression in different groups treated with different concentrations of VPA was examined (Fig. 4c, f), indicating that VPA exposure prohibited neural differentiation.

Furthermore, we assessed neural differentiation after the removal of VPA. Following treatment with VPA for 10 days, the organoids were cultured in the absence of VPA for another 10 days, allowing for neural differentiation. In comparison to the control group, the VPA-exposed group contained more neurons, characterized by a higher proportion of TUJ1 + cells (Fig. 4g). Additionally, the mRNA expression of TUJ1 was upregulated, confirming increased neuronal differentiation of the VPA-exposed group (Fig. 4h). This finding revealed that more neurons had differentiated from NPCs in organoids whose exposure to VPA was removed, suggesting the long-lasting effects of VPA exposure on neurogenesis.

In vivo, the development of higher cognitive function in the cerebral cortex depends on neural maturation to form functional neural networks following neurogenesis and differentiation. Two important neuronal subtypes, excitatory and inhibitory neurons, are involved in this process. To examine neural maturation into excitatory and inhibitory neurons, cortical organoids were exposed to VPA for 10 days and then cultured in the absence of VPA for another 10 days, allowing advanced maturation. As shown in Fig. 4i, j, VPA increased the number of excitatory and inhibitory neurons, as shown by the upregulated expression of vGLUT1 and GABA (GAD67), at both the protein and mRNA levels, which was determined by immunostaining and real-time PCR, respectively. All the results above showed that VPA exposure impaired neurogenesis in cortical organoids, accompanied by enhanced NPC pools, delayed neuron differentiation and altered neural maturation.

### Impaired forebrain regionalization and cortical organization in organoids exposed to VPA

**Fig. 5:**
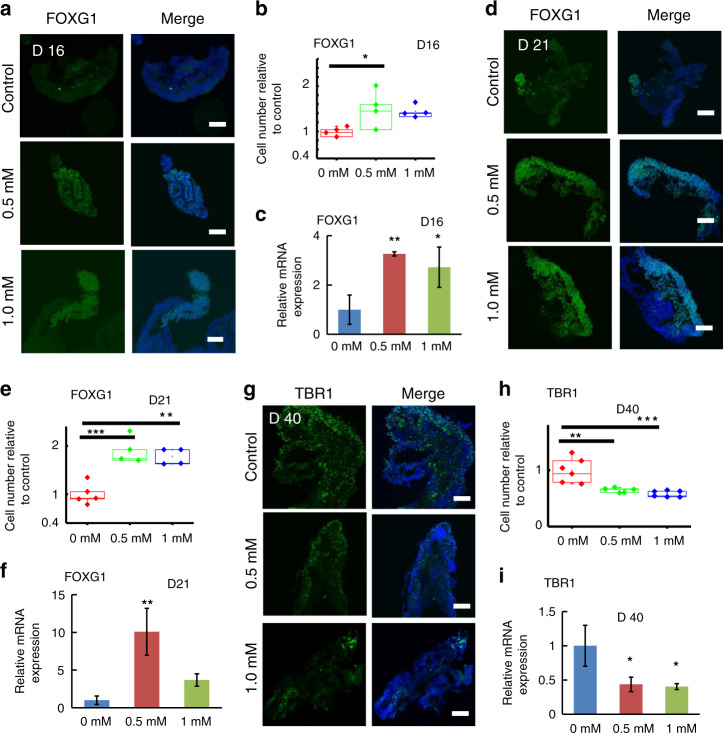
VPA exposure altered forebrain organization and cortical development in cortical organoids. **a**, **d** The expression of a forebrain marker (FOXG1) was detected by immunohistochemical staining. FOXG1 + cells were quantified based on immunofluorescence images. Four or five images were randomly selected for quantification on days 16 (**b**) and 21 (**e**). **c**, **f** The mRNA level of a forebrain marker (FOXG1) in the cortical organoids exposed to different concentrations of VPA on day 16 (**c**) and day 21 (**f**) was determined by real-time PCR. β-Actin acted as a normalization control gene. **g** Pre-plate neuron TBR1 expression in cortical organoids exposed to different concentrations of VPA for 5 days was identified by immunohistochemical staining. **h** Quantification of TBR1 + cells in VPA-exposed cortical organoids. Six random images were selected for quantitative analysis. **i** Relative mRNA expression of TBR1 by quantitative real-time PCR demonstrated that VPA exposure upregulated the expression of pre-plate TBR1. All data are the means of three replicates ± SDs. At least 10 random cortical organoids generated on one micropillar chip in each group were examined for quantitative analysis. In addition, three replicate chips for each group were analyzed in each independent experiment. Moreover, more than eight random tissue colonies for each sample were analyzed by immunohistochemical staining. The data were analyzed using a one-way ANOVA with Bonferroni post-test (*p < 0.05; **p < 0.01; ***p < 0.001; ****p < 0.0001). Scale bar = 100 μm.

As cortical organoids mainly recapitulate the forebrain, we examined the influence of VPA on development of the forebrain. FOXG1 is a typical marker of the forebrain; therefore, we next explored FOXG1 expression at the mRNA and protein levels after treatment with VPA at different concentrations for 5 and 10 days. As depicted in Fig. 5, VPA significantly accelerated the proliferation of FOXG1 + cells compared with that in the control group, as shown by immunohistochemical analysis (Fig. 5a, d) and quantification (Fig. 5b, e). Meanwhile, analysis of VPA-induced changes in brain regional development at the mRNA level was conducted by quantitative real-time PCR, and gene transcripts of the forebrain (FOXG1) were significantly upregulated (Fig. 5c, f). These data implied that VPA triggered dysfunction in the development of forebrain populations in organoids.

As cortical organoids exhibit an in vivo-like layered cortex, the effects of VPA on cortical organization were next explored. Cortical organoids were exposed to VPA at different concentrations from day 35 to day 40, during which initial cortical organization started. With VPA treatment (0.5 and 1 mM), cortical organoids exhibited markedly reduced TBR1 expression, as shown by analysis of immunohistochemical images (Fig. 5g) and a qualitative assay (Fig. 5h). Additionally, the mRNA expression of TBR1 was examined by quantitative real-time PCR, which further verified the reduced levels of TBR1 in VPA-exposed organoids (Fig. 5i). Combined, these results showed that VPA impaired forebrain regionalization and cortical organization in organoids.

### Transcriptome analysis of VPA-exposed cortical organoids

**Fig. 6:**
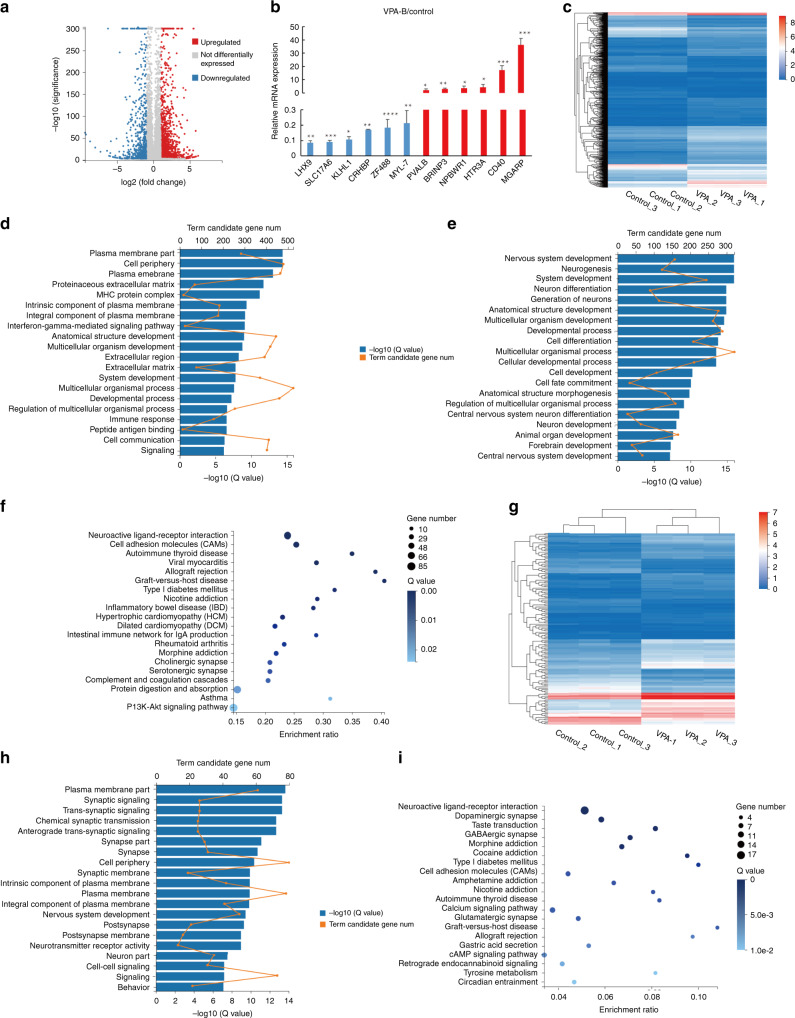
Transcriptomic analysis of cortical organoids exposed to VPA. **a** A volcano plot shows the DEGs in VPA-exposed cortical organoids compared with control organoids. In total, 2026 genes, including 1323 upregulated genes (in red) and 703 downregulated genes (in blue), exhibited significantly differential expression. **b** qRT-PCR validation of selected DEGs identified by RNA-seq. “/” indicates the relative value. Data are the mean ± SD. **c** Hierarchical clustering heat map of DEGs between the VPA-exposed group and the control group. **d** GO enrichment analysis of the 1323 upregulated genes. The *x*-axis indicates the term candidate gene number (yellow), the *x*-axis (lower) indicates the −log (*Q* value) (blue), and the *y*-axis indicates GO terms. **e** GO enrichment analysis of 703 downregulated genes. The *x*-axis indicates the term candidate gene number (yellow), the *x*-axis (lower) indicates the −log (*Q* value) (blue), and the *y*-axis indicates GO terms. **f** KEGG enrichment analysis of DEG genes. The *x*-axis indicates the enrichment ratio, and the *y*-axis indicates the KEGG terms. The size of the circle represents the gene number, and the color of the circle indicates the value (adjusted *P*-value). **g** Hierarchical clustering heat map of DEGs related to autism in the VPA-exposed group. **h** GO enrichment analysis of autism-related genes. The *x*-axis indicates the term candidate gene number (yellow), the *x*-axis (below) indicates the −log (*Q* value) (blue), and the *y*-axis indicates the GO terms. (**i**). KEGG enrichment analysis of autism-related genes among the DEGs. The *x*-axis indicates the enrichment ratio, and the *y*-axis indicates the KEGG terms. The size of the circle represents the gene number, and the color of the circle indicates the value (adjusted *P*-value). At least random 10 cortical organoids generated on one micropillar chip in each group were examined for RNA-seq. In addition, three replicate chips for each group were analyzed in each independent experiment.

To obtain a global view of gene expression in cortical organoids exposed to VPA, RNA-seq was performed. As shown by the results above, cortical organoids exposed to 0.5 mM VPA were always dominantly altered; therefore, organoids treated with 0.5 mM VPA were prepared for RNA-Seq analysis. We identified 7418 DEGs, including 4297 upregulated and 3121 downregulated genes (corrected *p* < 0.05, FDR), in the VPA-exposed group compared to the control group (Supplementary Table 1). For differential gene expression analysis, the *P*-value threshold after correction for multiple testing (adjusted *p*-value) was set at 0.01 and used with a threshold of a 2-fold change in mRNA expression. By these criteria, 2026 genes showed altered expression levels; among these genes, 1323 were upregulated, while 703 were downregulated, as demonstrated in the volcano plot (Fig. 6a). We further quantitated the expression of the most significantly upregulated and downregulated genes that regulate brain development by real-time PCR. These genes were significantly altered in VPA-exposed cortical organoids (Fig. 6b), consistent with the results of RNA-seq. Among these DEGs were some new genes, including KLHL1, LHX9, and MGARP, which were not previously reported in VPA-exposed brains. The actin-binding protein Kelch-like 1 (KLHL1) can modulate voltage-gated calcium channels in vitro^24^ and is potentially correlated with locomotory behavior, walking behavior, and neurogenesis. LHX9, expressed in the developing forebrain, is a member of the LIM homobox gene family^25^. It controls pineal gland development and prevents hydrocephalus^26^, which was found to be tightly associated with brain disorders^27^. MGARP negatively regulates neocortical development in mice and mitochondrial distribution and motility in neocortical neurons^28^_._ However, although these three genes are related to the nervous system, whether they are involved in the pathogenesis of neurological disease remains to be determined.

Hierarchical clustering of these genes demonstrated significant differences between the VPA-exposed and control groups (Fig. 6c). GO analysis revealed that the genes most affected by VPA are involved in the plasma membrane, cell periphery, plasma membrane, extracellular matrix and nervous system development, neurogenesis, neuron differentiation, and the generation of neurons (Fig. 6d, e). KEGG analysis showed that the top 20 pathways enriched in the DEGs are pathways related to neuroactive ligand–receptor interactions, CAMs and their participation in inflammation, the immune response, autoimmune diseases and cardiovascular diseases (Fig. 6f). The results indicated that the above signaling pathways might be involved in VPA-induced adverse neurodevelopmental outcomes.

Strikingly, we also found 144 autism-related risk genes that were changed in the VPA-exposed group compared with the control group. Hierarchical clustering of autism-related risk genes demonstrated significant differences between their expression in the VPA-exposed and control groups (Fig. 6g). GO enrichment analysis showed that these genes are related to synaptic function and the plasma membrane (Fig. 6h). As shown in Fig. 6i, these genes are enriched in neurodevelopmental pathways associated with dopaminergic, GABAergic, and glutamatergic synapses. The synapse is a specialized junction responsible of communication between neurons, and synaptic plasticity is the basis of learning and memory. Therefore, this finding indicated that VPA might induce extensive defects in learning and memory. In addition, other pathways involved in the autoimmune system were identified, suggesting the role of VPA in impairing the immune system. These results indicated that significant alterations in the expression of these genes in VPA-exposed cortical organoids might be associated with autism.

**Fig. 7:**
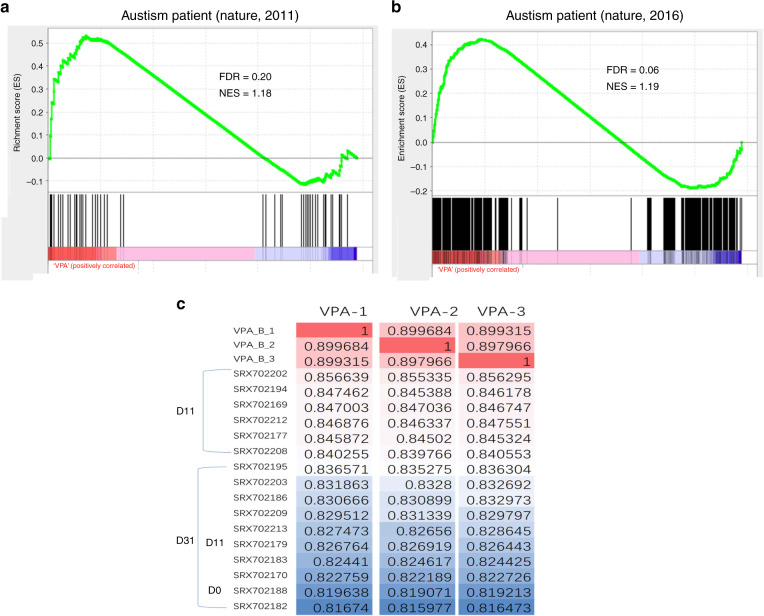
Comparison of transcriptome data from VPA-exposed cortical organoids, postmortem brain tissue from patients with autism and autism patient-derived cortical organoids by GSEA and correlation analysis. **a**, **b** The similarity between the transcriptomes of VPA-exposed cortical organoids and the postmortem brains of patients with autism previously published in 2011 and 2016. The normalized enrichment score (NES) indicates the enrichment magnitude, and the false discovery rate (FDR) shows the statistical significance. **c** Correlation coefficient analysis of VPA-exposed cortical organoids and autism-derived cortical organoids. Higher values indicate closer relationships between VPA-exposed cortical organoids and autism-derived cortical organoids. SRX702202, SRX702169, SRX702212, SRX702194, SRX702177, SRX702208, SRX702179 indicate data from neural progenitors derived from the iPSCs of patients with ASD at D11. SRX702195, SRX702203, SRX702209, SRX702186, SRX702213, SRX702183, SRX702170, SRX702182 indicate data from neural progenitors derived from the iPSCs of patients with ASD at D31. SRX702188 indicates data from neural progenitors derived from the iPSCs of patients with ASD at D0.

More importantly, we compared transcriptome data from VPA-exposed cortical organoids, postmortem ASD brain tissue^29,30^ and ASD hiPSC-derived cortical organoids^31^. First, we compared the transcriptomes of the postmortem human brain and VPA-exposed cortical organoids by gene set enrichment analysis (GSEA). GSEA of the two datasets indicated that the transcriptomes of VPA-exposed cortical organoids and the postmortem ASD human brain were very similar (Fig. 7a, b). Next, the transcriptomes of VPA-exposed cortical organoids and ASD-derived cortical organoids at three time points (D0, D11, D31) were compared^31^. This comparison showed that the transcriptome of VPA-exposed cortical organoids was most similar to that of the ASD-derived cortical organoids, but the VPA-exposed cortical organoid transcriptome was also significantly correlated with that of ASD-derived organoids on day 11 (Fig. 7c). These results indicated that the impaired neural development observed in cortical organoids exposed to VPA might contribute to postnatal neural disorders in ASD.

## Discussion

In this study, we present a human cortical organoid-on-a-chip model that allows us to probe impaired neurodevelopment after prenatal VPA exposure at early gestation for the first time. The cortical organoid-on-a-chip system facilitates the differentiation and self-organization of hiPSCs into cortical organoids in a simple and efficient way. The brain organoids recapitulated critical features of the developing human brain based on analysis of neural differentiation, forebrain regionalization, and cortical organization. After exposure to VPA, cortical organoids exhibited an increased NPC pool, altered neuronal differentiation and maturation, and disrupted forebrain and cortical regionalization. These alterations might lead to neural dysfunctions in individuals with neural disorders. Moreover, a series of genes and pathways related to neural development showed marked altered regulation in VPA-exposed organoids by transcriptome analysis. Notably, comparison of the transcriptomes of VPA-exposed cortical organoids, postmortem brain samples and autism-derived cortical organoids showed the strong similarity between VPA-exposed cortical organoids and autism samples. These results suggest that neurodevelopmental impairment in VPA-exposed cortical organoids contributes to postnatal neural disorders in ASD.

With VPA treatment, cortical organoids exhibited an enhanced NPC pool and delayed neural differentiation. Due to the proliferation and differentiation of NPCs, the enhanced stem cell pool produced more neurons after the removal of VPA, in turn increasing the number of neurons. These findings are consistent with the clinical observation of the increase number of neurons in autistic patients^32^. These findings also indicate that prenatal exposure of the fetal brain to VPA may increase the risk of autism in offspring. Moreover, VPA promoted the differentiation of excitatory and inhibitory neurons, as shown by the increased expression of vGLUT1 and GABA (GAD67) (Fig. 4). The impairment of neural differentiation might contribute to an imbalance between excitatory and inhibitory neurons, as has been observed in animal models. Additionally, VPA exposure altered expression of the forebrain marker FOXG1, which has not been observed in animal models, and the pre-plate cortical marker TBR1. FOXG1 is involved in the modulation of brain size and may modulate the social disability component of the autism phenotype^33,34^. Herein, FOXG1 was overexpressed in cortical organoids exposed to VPA, suggesting impaired regulation of brain size. Abnormal brain size is a typical biomarker (clinical feature) of macrocephaly or autism. TBR1 acts as an essential regulator of gene expression during cortical neurogenesis^35^. Previous reports showed that the downregulated expression of TBR1 stimulated the expression of autism-related genes, potentially leading to the occurrence of autism^36,37^. Together, our findings revealed that VPA exposure might lead to impaired neurogenesis during fetal brain development and thus potentially elicits the onset of brain disorders, such as autism, after birth.

Analysis of gene expression at the mRNA level revealed significant alterations in gene expression in VPA-exposed cortical organoids, and DEGs were shown to be tightly related to the occurrence of neurodevelopmental dysfunction. In particular, some new genes, including KLHL1, LHX9, and MGARP, that have not been reported in traditional animal models and cell culture systems were identified. KLHL1 is correlated with locomotory behavior and walking behavior. Its dysfunction might contribute to postnatal defects in behavior in neurodevelopmental disorders, such as autism, that were observed after prenatal VPA exposure^24^. LHX9 in the thalamus is essential for the modulation of processes such as sleeping, alertness and consciousness^25^. The downregulated LHX9 expression observed in VPA-exposed cortical organoids might provide new evidence to explain the disruption of sleep in individuals with psychiatric and neurodegenerative disease such as autism^38^. In addition, MGARP regulates mitochondrial distribution and motility in neocortical neurons. Previous studies have demonstrated mitochondrial dysfunction in patients with neurodegenerative diseases^39^ and autism^40^. Thus, the upregulation of MGARP in VPA-exposed cortical organoids could explain mitochondrial impairment observed in neurodevelopmental disorders.

In addition, GO analysis of upregulated genes in VPA-exposed cortical organoids showed that these genes encode extracellular matrix proteins (Fig. 6d) that are essential for the normal function of the cortex^41^. KEGG analysis revealed that the neuroactive ligand–receptor interaction, synapse function and plasticity pathways, which are tightly related to neural functions including learning and memory, were highly enriched in the DEGs^42^. Additionally, several signaling pathways associated with type 1 diabetes, inflammatory bowel disease and thyroid disease were significantly enriched in DEGs following VPA exposure. These pathways were previously reported to be altered in neurological disease, suggesting the close association between these diseases and neural disorders. In this work, a high proportion of DEGs in VPA-exposed organoids compared with control organoids are related to circadian entrainment. This is consistent with the clinical observation that patients with autism often have problems with sleep, memory, and timing. Taken together, these results reflect genome-wide changes related to neurodevelopmental disorders (e.g., autism) in VPA-exposed cortical organoids. In particular, VPA-exposed organoids were shown to be very similar to the postmortem ASD brain through GSEA. In addition, the transcriptomes of the VPA-exposed organoids and autism-derived organoids were demonstrated to be very similar via correlation analysis. We assume that VPA exposure impairs neurogenesis at the early stage of brain development, leading to autistic tendencies to some extent. This may contribute to the outcome of behavioral disorders in infancy and childhood.

Collectively, the established cortical organoid-on-a-chip model serves as a valuable platform and offers new evidence associating autism-like neurodevelopment disorder with prenatal VPA exposure. This technique not only allows the simple generation of an array of cortical organoids from hiPSCs with a uniform size but also facilitates the extended study of brain development and neurological diseases and predictions of the effects of various prenatal environmental insults during early gestation.

## Materials and methods

### Fabrication of micropillar chips

According to a previous article^43^, micropillar chips were made of polydimethylsiloxane (PDMS, Dow Corning Corp) via soft lithography. The microarray chips included two PDMS layers, among which the bottom layer consisted of patterned micropillars with a diameter and height of 1 mm, while the top layer consisted of a ring structure (with the dimension of a 24-well plate). The micropillar chips were produced according to the steps below. First, PDMS monomer was mixed with a curing agent (184 Silicone Elastomer, Dow Corning Corp) at the ratio of 10:1 by mass and degassed to remove air bubbles. The polymer was cured in an oven for 40–60 min at 80 °C. The PDMS layer was then gently peeled from the mold.

### hiPSC culture and maintenance

According to a previous article^44^, briefly, hiPSCs derived from the skin fibroblasts of a human male, a kind gift from Dr. Ning Sun, were maintained in mTeSR1 medium (Stemcell Technologies) on plates precoated with Matrigel (BD) for ~5 days. When the hiPSCs were 80–85% confluent, they were digested with Accutase (Sigma) and formed small colonies (~200 μm in size). Finally, mTeSR1 medium containing the ROCK inhibitor Y27632 at 10 μM was used to resuspend hiPSC pellets, which were then seeded on new Matrigel-coated plates and maintained in an incubator for an hour. After 1 h, the mTeSR1 medium containing the ROCK inhibitor Y27632 was replaced with mTeSR1 medium alone.

### Formation of cortical organoids on the chip

Cortical organoids initiate from EBs which are 3D hiPSC-derived multicellular clusters. To form EBs, single hiPSCs were resuspended in mTeSR1 medium containing the ROCK inhibitor Y27632 at 15 μM, seeded on micropillar chips, and maintained for 1 day. On the 2nd day, the medium was replaced with KSR medium supplemented with additional factors. Knockout serum replacement (KSR) medium included the following ingredients: 20% KSR (Invitrogen), 80% DMEM/F12 medium (Invitrogen), 1% GlutaMAX (Invitrogen), 1% minimum essential media-nonessential amino acids (MEM-NEAA, Invitrogen), penicillin–streptomycin (Sigma), 0.2 mM 2-mercaptoethanol (Sigma-Aldrich), the ROCK inhibitor Y27632 at 15 μM and 4 ng ml^−1^ basic fibroblast growth factor (bFGF, Peprotech). In addition to the reagents above, the medium also contained two inhibitors, the AMPK inhibitor dorsomorphin (Selleck) and the TGF-β inhibitor A83–01 (Sigma). Dorsomorphin, A83–01 and Y27632 were added to the KSR medium for the first 3 days. From day 4 to day 6, the KSR medium contained dorsomorphin/A83–01 and bFGF. On day 6, the KSR medium was replaced with neural induction medium (NIM) consisting of DMEM/F12, 1% MEM-NEAA, 1% N2 supplement (Invitrogen), 1% penicillin–streptomycin, 1% GlutaMAX, and 1 μg ml^−1^ heparin (Sigma). From day 6 to day 9, the NIM contained bFGF. From day 9 to day 11, the cortical organoids were cultured in NIM.

### Exposure of cortical organoids to VPA

To probe the effects of VPA on developing cortical organoids, randomly selected developing cortical organoids were exposed to VPA at different concentrations for 5 days and 10 days. At least 10 cortical organoids that had been generated on one micropillar chip in each group were randomly examined for quantitative analysis (e.g., real-time PCR). The medium was changed every other day. The NDM medium contained equal amounts of DMEM/F12 and neurobasal medium and 0.5% N2 supplement, 1% B27 supplement (50x, Gibco), 0.1 mM beta-mercaptoethanol, 1% GlutaMAX, 0.5% MEM-NEAA and 1% penicillin–streptomycin. Because of the potential for VPA to be absorbed by PDMS, we used VPA in its salt form, sodium valproate, which has a high solubility in water (50 g/L)^45^. In addition, the logarithm of the distribution coefficient of sodium valproate is <2.47; therefore, the potential impact of VPA absorption by PDMS would be negligible^46^. Sodium valproate was dissolved in ddH_2_O at a concentration of 300 mM as a stock solution.

### RNA extraction and quantitative real-time PCR (qRT-PCR)

Briefly, according to a previous article, total mRNA was extracted from organoids and hiPSCs with RNAiso Plus, and the concentrations were measured with a NanoDrop (Thermo Fisher Scientific) and adjusted to 69 ng/µl. Reverse transcription was carried out in samples containing reverse transcription reagent and mRNA at 1:5 using PrimeScript RT Master Mix (Takara). The cDNA was amplified using Ex Taq DNA polymerase (Takara) under the following reaction conditions: 35 cycles of denaturation at 94 °C for 1 min, annealing at 58 °C for 45 s, and extension at 72 °C for 30 s. The primers used for this analysis are listed in Supplementary Table 2.

### Tissue cryosection and immunohistochemistry

According to a previous article, briefly, cortical organoids were fixed in 4% paraformaldehyde overnight at 4 °C and then dehydrated by incubation with 30% sucrose overnight at 4 °C. The cortical organoids were then embedded in OCT compound (Sakura) and cryosectioned at 10 mm with a cryostat (Leica). For immunohistochemistry, frozen sections were washed with PBS before permeabilization with 0.2% Triton X-100 for 5 min at room temperature. The sections were blocked with 10% blocking serum (Solarbio, SL1) for 1 h at room temperature and then incubated with primary antibodies against the following in blocking solution at the corresponding dilutions: active caspase 3 (rabbit, 1:250, Abcam, ab32042), FOXG1 (rabbit, Abcam, ab196868, 1:500), NESTIN (mouse, Santa Cruz, sc-20978, 1:400), PAX6 (rabbit, BioLegend, PRB-278P, 1:300), SOX2 (rabbit, Cell Signaling, 3579, 1:400), TUJ1 (mouse, BioLegend, 801201, 1:500), PAX2 (mouse, Abnova, H00005076-M01, 1:400), CTIP2 (rat, Abcam ab18465, 1:500), TBR1 (rabbit, Abcam, ab31940, 1:200), vGLUT1 (mouse, Millipore, MAB5502, 1:400), GABA (rabbit, Sigma, A2052, 1:400), and ISL1 (mouse, Thermo, MA5-15515, 1:100). The secondary antibody used was Alexa Fluor 488- or 594-conjugated anti-donkey (1:500), and the sections were incubated with secondary antibody for 1 h at room temperature before being washed with PBS and subsequent microscopic inspection.

### Statistical analysis

Data are expressed as the means ± SDs. The data were analyzed by t-test or using one-way ANOVA with Bonferroni post-test. Significance levels were indicated as follows: *p < 0.05; **p < 0.01; ***p < 0.001; ****p < 0.0001. Sample sizes were indicated in the figure legends. Immunostaining images were quantified with Image-Pro Plus 6.0. In addition, the data were processed with Excel, Origin8, and GraphPad Prism 5.

### RNA sequencing

RNA sequencing of VPA-exposed brain organoids and control organoids was performed at day 18. Total mRNA was extracted from the organoids with RNAiso Plus and then dissolved in DEPC-treated water. Oligo (dT)-conjugated magnetic beads were used to purify mRNA, which was fragmented into small pieces in fragment buffer at the appropriate temperature. First-strand cDNA was generated by PCR with a first-strand reaction kit, and second-strand cDNA was generated as well. The reaction product was purified by magnetic beads, after which A-Tailing Mix and RNA index adapters were added by incubation for end repair. The cDNA fragments with adapters were amplified by PCR, and the products were purified with AMPure XP beads. The library was assessed for quality and quantity using an Agilent 2100 bioanalyzer. Then, DSN treatment was carried out. The DSN-treated library was assessed for quality with two methods to ensure the high quality of the sequencing data; the fragment size distribution was checked using the Agilent 2100 bioanalyzer, and the library was quantified using real-time quantitative PCR (qPCR). The qualified library was amplified on cBot to generate the cluster on the flow cell. In addition, the amplified flow cell was single paired-end sequenced on a HiSeq4000 or HiSeq X Ten platform (BGI Shenzhen, China). The RNA-seq data reported in this paper are accessible in the SRA with the accession code PRJNA544167.

## Supplementary information

Table S1

Table S2

Figure S1

## Data Availability

All data needed to reach the conclusions in this paper are present in the paper and/or the Supplementary Materials. In addition, the raw RNA-Seq: BioProject data are available at the SRA under the accession code PRJNA544167.
